# From Smoking to Cancers: Novel Targets to Neuronal Nicotinic Acetylcholine Receptors

**DOI:** 10.1155/2011/693424

**Published:** 2011-04-28

**Authors:** Chia-Hwa Lee, Chih-Hsiung Wu, Yuan-Soon Ho

**Affiliations:** ^1^Graduate Institute of Medical Sciences, Taipei Medical University, Taipei 110, Taiwan; ^2^Department of Surgery, School of Medicine, Taipei Medical University, Shuang Ho Hospital, Taipei 235, Taiwan; ^3^Department of Laboratory Medicine, Taipei Medical University Hospital, Taipei 110, Taiwan; ^4^Center of Excellence for Cancer Research, Taipei Medical University, Taipei 110, Taiwan

## Abstract

Cigarette smoking bears a strong etiological association with many neovascularization-related diseases, including cancer, cardiovascular disease, and age-related macular degeneration. Cigarette smoke is a complex mixture of many compounds, including nicotine, which is the major active and addictive component of tobacco. Nicotine and its specific metabolized carcinogens directly bind to nicotinic acetylcholine receptors (nAChRs) on cell membranes and trigger the nAChR signal cascade. The nAChRs were originally thought to be ligand-gated ion channels that modulate physiological processes ranging from neurotransmission to cancer signaling. For several decades, the nAChRs served as a prototypic molecule for neurotransmitter receptors; however, they are now important therapeutic targets for various diseases, including Alzheimer's and Parkinson's diseases, schizophrenia, and even cancer. This paper describes recent advances in our understanding of the assembly, activity, and biological functions of nicotinic receptors, as well as developments in the therapeutic application of nicotinic receptor ligands.

## 1. Introduction

The impact of tobacco use on mortality and morbidity is well known. As far back as 1982, the Surgeon General of the United States Public Health Service has concluded that cigarette smoking is the major single cause of cancer mortality in the United States. Recently, the World Health Organization (WHO) reported in 2010 that almost one billion people and 250 million women are daily smokers. The tobacco epidemic kills 5.4 million people in average per year from lung cancer, heart disease, and other illnesses, and approximately 650,000 of these deaths are caused by second-hand smoke. If this smoking trend continues, there will be more than 8 million deaths every year, with more than 80% of tobacco-related deaths in developing countries by 2030. Consequently, tobacco will kill a billion people due to smoking-related disease during this century, with tobacco use-related cancers being one of the main causes of death. 

Tobacco use is by far the most widespread factor causing exposure to known carcinogens and death from cancer and is therefore a model for understanding mechanisms of cancer induction. A causal relationship was reported between active smoking and cardiovascular diseases, respiratory diseases, reproductive disorders, and several types of cancers, including cancers of the lung, bladder, cervix, esophagus, kidney, larynx, mouth, pancreas, stomach, and leukemia [1]. Although it might seem obvious that carcinogens associated with the use of tobacco products have caused numerous cancers, the effects of cancer genes, protein complexes, cellular circuitry, and signal transduction pathways are often overlooked. 

According to the report from the International Agency for Research on Cancer in 2010, cigarette smoke contains a diverse array of 4,000 chemicals, 250 of which are known to be harmful, and more than 60 known carcinogens have been detected in mainstream cigarette smoke, and most of the same carcinogens are also present in second-hand smoke. The most potent of these carcinogens are polycyclic aromatic hydrocarbons and nicotine-specific metabolites, such as 4-(methylnitrosamino)-1-(3-pyridyl)-1-butanone (NNK) and N-nitrosonornicotine (NNN). These nitrosamines form DNA adducts cause mutations that lead to cancer [2]. DNA adducts have been proposed as potential markers of exposure to tobacco carcinogens, and these markers may help provide an integrated measure of carcinogen exposure relevant to individual cancer risk assessment. The adduct levels are generally higher in lung tissues of smokers than those of nonsmokers while studies using blood DNA have produced mixed results. In the following sections, we review evidence showing how nicotine or nicotine-specific metabolic nitrosamines, NNK or NNN, promote cancer development through the physical interaction with nicotinic acetylcholine receptors (nAChRs).

## 2. Genomewide Association of nAChRs with Lung Cancer

Many studies have pointed out that the binding of exogenous nicotine, NNK, NNN, and acetylcholine to nAChRs, respectively, will stimulate the growth of both small cell lung carcinomas (SCLCs) and nonsmall cell lung carcinomas (NSCLCs) [3]. Two similar studies also showed that the autocrine interaction of acetylcholine (Ach) and estrogen with the nAChR will stimulate SCLC and breast cancer cell proliferation [4–6]. To identify genetic factors involved in smoking-mediated cancer risk, a genomewide association study of 317, 139 single-nucleotide polymorphisms was recently performed using DNA from 1,989 lung cancer patients and 2,625 control subjects from six central European countries [7]. A locus in the 15q25 chromosome region was found to be strongly associated with lung cancer [8]. Interestingly, this region contains several genes, including three nAChR subunits (*CHRNA5*, *CHRNA3*, and *CHRNB4, *encoding the *α*5, *α*3, and *β*4 subunits, resp.) that are predominantly expressed in neurons and other tissues (particularly alveolar epithelial cells, pulmonary neuroendocrine cells, and lung cancer cell lines) [7, 9, 10]. Previous studies have also suggested that *N*′*-nitrosonornicotine* and nitrosamines may facilitate neoplastic transformation by stimulating angiogenesis and tumor growth mediated through their interaction with nicotinic acetylcholine receptors [11, 12]. The activation of these receptors can also be inhibited by nicotine receptor antagonists, which confirms that nAChRs play important roles in disease development and implies possible chemoprevention opportunities for lung cancer [13]. Therefore, further analyses of multiple diverse populations will be required to confirm this locus and to identify additional lung cancer susceptibility.

## 3. Nicotinic Acetylcholine Receptor Structure

The nicotinic acetylcholine receptors (nAChRs) belong to the superfamily of the Cys-loop ligand-gated ion channels (LGICs), which also include the GABA, glycine, and 5-HT3 receptors. They are formed by the assembly of five transmembrane subunits selected from a pool of 17 homologous polypeptides (*α*1–10, *β*1–4, *γ*, *δ*, and *ε*). There are many nAChR subtypes, each consisting of a specific combination of subunits, which mediate diverse physiological functions. These receptors are widely expressed in the central nervous system, and, in the periphery, they mediate synaptic transmission at neuromuscular junctions and ganglia. Recently, the cDNAs for all types of nAChR subunits have been cloned from neuronal and nonneuronal cells, such as keratinocytes, epithelia, and macrophages, which encompass the main domains of the ligand-binding sites.

Based on the different ligand-binding properties of these nAChRs, the nAChRs are divided into two main classes: (1) the *α*-bungarotoxin-(*α*-Bgtx-) binding nAChRs, which are mainly homopentamers of *α*7, *α*8, or *α*9 subunits; (*2*) nAChRs, which do not bind *α*-Bgtx but consist of the *α*2–*α*6 and *β*2–*β*4 subunits, exist only as heteropentamers and bind agonists with high affinity [14]. The presence of a certain subunit can affect the localization, biophysical, functional, and pharmacological properties of the nAChRs, as well as the regulation of the expression of the nAChR subtype at the developmental or adult stage in some specific cells. The absence of a subunit may lead to the compensatory upregulation of other subtypes [15].

Because nAChR subunits exhibit a high degree of evolutionary conservation, studies of high-resolution X-ray crystallographic and electron microscopic analyses of proteins related to nAChRs have provided considerable insight into how structure imparts functional similarities and differences among all nAChRs.

Multiple nAChR subunit compositions are expressed in the central and peripheral nervous system, but the most represented receptors are *α*4*β*2 and *α*7 in the brain and *α*3*β*4 in the peripheral nervous system. In these nAChRs, *α*4*β*2-composed nAChRs have the highest affinity to nicotine [16, 17]. In addition, only the *α*4 and *β*2 subunits are found on GABA-Aergic neurons [18]. Another study pointed out that *α*4*β*2 levels can be upregulated by proinflammation cytokines, such as TNF-*α* [19] through p38-MAPK signaling pathways. This important discovery reveals the complexity of the interaction network between nAChRs and the inflammation factors. By contrast, compared to *α*4*β*2 nAChR, *α*7 homopentameric nAChR is the most well-known and investigated type of nAChR. Receptors composed of *α*7 subunits are known to desensitize rapidly and to have a high Ca2^+^ : Na^+^ permeability ratio that exceeds that of the glutamate NMDA receptor [20–22] and the 3-4 : 1 ratio of most other nAChRs. The signaling pathway encourages scientists to look further into carcinogenetic mechanisms underlying *α*7-nAChRs-related lung [23], bladder [24], and colon cancers [25], as well as *α*9-nAChRs in breast cancers [26–28]. In fact, some receptors (such as *α*7, *α*9, and *α*10) have highly specialized functions including those pertaining to the regulation of signaling mechanisms used by sensory epithelia and other nonneuronal cell types [29].

## 4. nAChR Signaling Pathways

Cigarette smoking has a strong etiological association with the development and progression of several types of cancers, cardiovascular disease, diabetic retinopathy, and age-related macular degeneration. Nicotine is the major active and addictive component of cigarette smoke. Previous studies demonstrated that the average plasma nicotine concentration of active smokers is about 100 nM to 1 *μ*M [30, 31]. In addition to active cigarette smoking, exposure to second-hand smoke is another mode of nicotine exposure [32]. When the biological levels of nicotine associated with second-hand smoke exposure were measured, a positive correlation between second-hand smoke exposure and concentrations of nicotine in the body was found. To date, it is well known that the specific nicotine-metabolized, tobacco-specific carcinogenic nitrosamines NNK and NNN are strong mutagens associated with several cancers, including lung, bladder, colon, and breast cancers [33–38]. Through binding of several ligands and nAChRs, signaling transductions are able to activate and promote cell proliferation, migration, and metastasis in cancer cells. 

As the nAChRs are ligand-gated cationic channels, their different subtypes, such as neuronal nAChRs, are differentially permeable to calcium ions [39, 40]. The calcium permeability of homomeric receptors is significantly higher than heteromeric nAChRs [39, 40]. In particular, the *α*7-containing nAChRs are generally considered to be the most permeable receptors to calcium, and their activation can raise cytoplasmic calcium levels and trigger a series of calcium-dependent intracellular processes [39, 40]. Recent studies have demonstrated the presence of nAChRs in several nonneuronal, nonexcitable cells, including bronchial epithelium, endothelial cells, keratinocytes, immune cells, and vascular smooth muscle cells [15, 41, 42]. The presence of these receptors in nonneuronal cells seems to suggest that they have distinct functions well beyond neurotransmission [43–49]. 

Several convergent studies have indicated that the *α*7-nAChRs primarily mediate endothelial cell proliferation, invasion, and angiogenesis [50–55]. The presence of *α*7-nAChR inhibitors like methyllycaconitine (MLA) and *α*-bungarotoxin could reverse the proangiogenic effects of nicotine. However, it must be noted that both *α*-bungarotoxin and MLA also bind with high affinity to *α*9-nAChR. Therefore, there may be partial involvement of *α*9-nAChR in the proangiogenic effects of nicotine [56]. The involvement of nAChR subunits in nicotine-induced angiogenesis was further verified by siRNA techniques.

In general, nicotine induced cell proliferation, angiogenesis, migration, and apoptosis through nAChR-associated downstream signal transduction such as MAP Kinase, PI3-kinase/Akt, NF-*κ*B, and *β*-arrestin pathways [50–53, 57–60]. Through the above signal transduction, it is found that in nonneuronal tissues, nicotine induces the secretion of growth factors such as *β*FGF, TGF*α*, VEGF, PDGF [61], and the upregulation of the calpain family of proteins [62], COX-2 and VEGFR-2 [63]. Most intriguingly, both VEGF- and *β*FGF-induced human microvascular endothelial cell (HMVEC) migration and angiogenesis require nAChR activation [64]. 

Mechanistically, nicotine has been shown to induce activation of NF-*κ*B through the MAP kinase and PI3K/AKT signaling pathways, which promote survival, proliferation, and angiogenesis of endothelial cells [65]. Further study showed pharmacological dissection of nicotine's influence on cell cycle progression, apoptosis, and differentiation [43], and this indicates that *α*7-nAChRs expressed in keratinocytes are important. In addition, large-cell carcinoma, squamous-cell carcinoma, adenocarcinoma of small airway, and alveolar type II cell of origin, as well as immortalized large- and small-airway epithelial cells all confirmed that nicotine and NNK activate the PI3K-Akt pathway and NF-*κ*B, resulting in stimulation of proliferation and inhibition of chemotherapy-induced apoptosis [66, 67]. Recently, a study also demonstrated that AKT survival signals play an important role in the nicotine-mediated carcinogenic process in human breast cancer cells [28]. 

In addition, *β*-arrestin-1 and Src kinase also appear to be the key players in mediating the mitogenic effects of nicotine. The Src family of protein tyrosine kinases has been found to be a critical component of multiple receptor-mediated signaling pathways that regulate proliferation, survival, metastasis, and angiogenesis. Additionally, nicotine also promotes cancer cell invasion by inducing matrix metalloproteinases 2 and 9, as well as the expression of plasminogen activators (urokinase-type plasminogen activator and its receptor) through COX-2 and VEGFR regulation [63]. 

Taken together, nicotine promotes cell proliferation and tumor angiogenesis via the stimulation of nAChRs. Nicotinic receptor antagonists, such as mecamylamine and *α*-bungarotoxin, demonstrate potent therapeutic application. Therefore, the development of specific, potent nAChR analogs and antagonists could provide novel approaches for the treatment of neovascularization-related diseases including cancer, cardiovascular disease, and macular degeneration.

## 5. Smoking, nAChRs, and Cancer

Cigarette smoking bears a strong etiological association with cancers. To the best of our knowledge, smoking-induced transformation can be abstracted into two aspects: (1) among the mixture of cigarette smoking compounds, NNK and NNN play the role of initiators in carcinogenesis (indirect-acting carcinogens, the most important tumorigenesis model in lung and bladder cancers). In contrast, nicotine has been demonstrated as a cocarcinogenic factor by playing a promoter role of carcinogenesis in tobacco replacement therapies [68]. Nicotine and NNK are considered to be carcinogens that react to DNA, and most reports have proposed that the chemical properties of the resulting DNA adducts can cause diverse genetic changes known to exist in human cancers [69–71]. (2) Nicotine, NNN or NNK have strong abilities to upregulate nAChR expressions which promote signals cascade, all these events result in a strong feedback loop and cause enhancement of cancer cell proliferation, migration, and metastasis. Therefore, understanding the functional diversity of the nAChR in each tissue could offer useful and abundant prospects for the designing of the novel cancer therapeutics stratagem. 

Since the brain is the best organ to characterize the role of nAChR in the regulation of the neurotransmitter acetylcholine [72], the interaction of nicotine with nAChR subunits in the brain provides the basis for nicotine addiction. For decades, nAChRs were generally believed to exist only in the nervous system (neuronal nAChRs) and at neuromuscular junctions (muscle nAChRs). However, in the past 20 years, increasing studies have shown that nAChRs can also be expressed in oral [34], mechanosensory hair [73], and airway epithelium cells [74, 75], where they play different roles in normal cell development and function. Furthermore, recent studies have also shown evidence that nAChRs and their physiological ligands such as choline and acetylcholine are universally expressed in mammalian and, more importantly, in cancer cells [24–26, 76–80]. The first study that implicated nAChRs in cancer growth regulation was reported in 1989 [81], and in the following decades, many studies indicated that nAChRs are the key molecular and central regulators of a complex network of stimulatory and inhibitory neurotransmitters that govern the synthesis and release of growth [23, 26, 82, 83], angiogenic [58], metastasis [25], and even apoptosis [84–86] in cancer cells microenvironment. In addition, the nAChRs are also found to trigger intracellular signaling pathways in a cell-type-specific manner.

The expression of nAChRs in mammalian cells and their diverse regulatory functions suggest that the modulation of these receptors, owing to chronic exposure to tobacco constituents or other environmental and lifestyle factors, contribute to the development of cancer [1]. This hypothesis was supported by the discovery that the tobacco-specific carcinogenic nitrosamines NNK, and NNN are agonists of *α*7-nAchR and the heteromeric *αβ*nAChRs, respectively [87]; both these nitrosamines cause lung cancer in laboratory animals [88]. The affinity of NNK for *α*7-nAChR was found to be about 1,300-fold higher than for nicotine, whereas the affinity of NNN for the heteromeric *αβ*nAChRs was about 5,000-fold higher [34, 87]. Because of their high affinity for nAChRs, NNK and NNN, rather than nicotine, might be the actual ligands for nAChRs in the context of smoking tobacco. Therefore, many of the addictive, neuropsychological, and cancer-stimulating effects from smoking that are currently attributed to nicotine are probably caused by these nitrosamines. In support of this hypothesis, a study displayed that the binding of NNK to *α*7-nAChR causes an influx of Ca^2+^ into lung cells, and the resulting membrane depolarization activated voltage-gated Ca^2+^ channels [49]. These eventually upregulated nAChRs expression [27, 89]. These data demonstrated a strong positive feedback loop associated with nAChR signaling that eventually causes normal cells to step into precancerous phase of transformation. Although all nAChRs are cation channels, they regulate diverse functions in a cell-type-specific manner. This functional diversity is also reflected in cancers of different cellular origins. In the following sections, the two latest and most important nAChR-induced cancer formation models will be illustrated.

## 6. ***α***7-nAChRs and Lung Cancer

The presence of nAChRs in lung cancer cell lines has been well investigated since 1989-1990; the first report suggested that nicotine and NNK bound to nAChRs would stimulate the proliferation of human small cell lung cancer (SCLC) cells through autonomic nervous-system-dependent regulation of lung cancer cells [81, 90]. This study was then reviewed by Maneckjee and Minna in 1990 and 1994 who demonstrated a nicotine-induced reversal of apoptosis in response to opioids in SCLC and NSCLC cell lines [91, 92]. Another laboratory discovered that nicotine affects the proliferation of human SCLC cell lines by stimulating the release of serotonin, which acts as an autocrine growth factor in these cells [93]. In turn, these findings led to the hypothesis that human airway epithelial cells express all of the components required to synthesize and secrete members of the acetylcholine family and nAChR subtypes. 

Nicotine exposure induces the augmented expression of  *α*7-nAChRs, which causes an influx of Ca^2+^ and activates downstream signals, such as protein kinase C, Raf-1, extracellular-signal regulated kinase (ERK) 1/2, and c-Myc, leading to increases in cell proliferation, cancer cell migration, metastasis, or the inhibition of apoptosis. West and colleagues [66] suggested that redundant Akt activation by nicotine and its carcinogen derivative NNK could contribute to tobacco-related carcinogenesis in nonimmortalized human airway epithelial cells. In this study, normal human bronchial epithelial (NHBE) cell was forced to be transformed through nicotinic activation of Akt which alters epithelial cell growth characteristics. Dysregulated NHBE growth after nicotine administration is consistent with *in vivo* observations of active smokers in which increased proliferative indices were seen when compared with former smokers. Protection from prolonged serum-deprivation-induced apoptosis, conferred by nicotine was attenuated by LY294002 or by DH*β*E, and protection conferred by NNK, was attenuated by LY294002 or by *α*-BTX [66]. This study showed that, in addition to promoting cellular survival or transformation process, nAChR activation from nicotine or NNK-induced Akt signal is required for diminishing contact inhibition and cellular dependence on exogenous growth factors or extracellular matrix. It revealed that abundant *α*7-nAChR expression in human cancer cells could be selectively attenuated by specific antagonists. Recently, Schuller [94] also proved that NNK interacts with *α*7 nAChRs, resulting in the development of lung cancer. The signals involved in normal cell transformation might be due to a significant reversible upregulation of the *α*1, *α*5, and *α*7 subunits in human bronchial epithelial cells, when these cells were exposed to nicotine (100 nM) *in vitro* for 72 hours. Since studies have shown that *α*7 is the main nAChR subunit that mediates the proliferative effects of nicotine in lung cancer cells [33, 95–99], *α*7-nAChR might be a valuable molecular target specifically for lung cancer therapy [100–102].

## 7. ***α***9-nAChRs and Breast Cancer

The expression of estrogen receptors by breast cancer cells has provided a therapeutic target by using estrogen receptor antagonists, but their use contributes to an unfortunate stimulation of breast cancer development through the pharmacological use of estrogen, the ligand for the estrogen receptor. Estrogen was recently found to differentially modulate nAChR subtype [5], and the expression of nAChR by breast cancers may similarly provide a new target for breast cancer therapies, whereas nicotine, a ligand for nAChR, was found to have stimulated breast cancer growth.

The *α*9-nAChR is a known homopentamer that plays a central role in coordinating keratinocyte adhesion and motility during wound healing [4]. Lee et al. [26] showed that *α*9-nAChRs were found to be ubiquitously expressed in many epithelial, lung, and breast cancer cell lines, and that most of the same cell lines also expressed *α*5- and *α*10-nAChRs [26]. The *α*9-nAChRs were present in primary tumors and nonmalignant breast tissue obtained from patients; however, breast cancer cells had increased *α*9-nAChR expression compared with the surrounding normal tissues. Lee et al. used MDA-MB-231 breast cancer cells, in which *α*9-nAChR expression had been silenced, to show that lowering *α*9-nAChR expression would reduce proliferation and tumorigenic potential in both *in vitro* and *in vivo* assays. Cells with inducible *α*9-nAChR gene expression were also generated from normal breast epithelial cells (MCF-10A) that were transformed by nicotine or NNK treatments, and experiments showed that increased *α*9-nAChR expression *in vitro* enhanced proliferation and colony formation. Likewise, mice that were subcutaneously injected with nicotine-transformed MCF-10A cells that inducibly expressed increased levels of *α*9-nAChRs showed enhanced tumor xenograft volumes when exposed to nicotine. Several studies have reported that nicotine decreases the cytotoxicity of doxorubicin, promotes migration via a signaling cascade involving protein kinase C and cdc42, and induces the proliferation, invasion, and epithelial-mesenchymal transition of breast cancer cells [103–105]. These studies provided evidence that nAChR, more specifically *α*9-nAChR, might play a major role in breast carcinogenesis, just as *α*7-nAChR is often associated with lung cancer [66], which further supports epidemiological studies that have revealed an association between breast cancer and exposure to cigarette smoke [106]. In conclusion, all the above demonstrated that *α*9-nAChRs expression knockdown can indeed inhibit breast cancer cell growth, whereas overexpression of *α*9-nAChRs, accompanied with long-term treatment nicotine, causes normal breast epithelial cell transformation both *in vitro* and *in viv*o experimental studies.

## 8. Developing Drugs Targeted at nAChR

### 8.1. nAChRs Agonist

Recent studies have shown that nicotine is not only a harmful product in cigarettes, but it is also a therapeutic nAChRs stimulator that enhances wound healing in preclinical models [53, 58, 107, 108]. Notably, these studies were conducted in animal models, and no side effects of nAChR agonists or antagonists were reported. Several neurological diseases associated with aging have been linked to reduced angiogenesis in the brain, and changes in the levels of nAChR in vascular-related cells in Alzheimer's disease [109, 110], and this suggests that there could be a role for a nicotine-based therapy in neurological disorders. 

Many of the studied, clinically used drugs that target nAChRs are administered for months, resulting in long-term changes in receptor properties and/or number. Accordingly, these drugs can be divided into two categories: *α*7-nAChR or non-*α*7-nAChR target agents. There are many potential drugs targeting nAChRs, and most of them are agonists and can be applied to treatment of various nervous-system disorders. For example, GTS-21, TC-5619, or EVP-6124 can be used for schizophrenia therapies [111]. The major target disease for a cognition enhancer is Alzheimer's disease. In Alzheimer's brain tissue, cortical nAChRs (*α*4*β*2) are markedly reduced (>80%), reflecting the cholinergic deficits associated with Alzheimer's disease [112]. Pilot trials using nicotine patches have demonstrated improved attention in Alzheimer's disease patients [113]. Interestingly, pharmacoepidemiological studies have shown a reduced incidence of Alzheimer's disease in populations of individuals who have previously smoked [114]. The potential protective effects of (−)-nicotine in this neurodegenerative disease may be related to neuroprotective properties observed with nicotine and other nAChR activators in *in vitro* and *in vivo* experimental studies. To our knowledge, Alzheimer's disease-specific therapies are mainly agonists of *α*7-nAChR. For example, SSR-180711, MEM-3454/R-3487, MEM-63908/R-4996, AZD-0328, and S-24795 are used. The *α*4*β*2-nAChR agonists are TC-1734 and S-38232; these drugs have shown promises in preclinical cognition models [111]. Other related drugs that act on the *α*4*β*2-nAChR can also be applied to smoking cessation, attention-deficit hyperactivity disorder, cognitive dysfunction, and depression [111].

### 8.2. nAChRs Antagonist

Neurotoxins are commonly used to distinguish between neuronal nAChR receptor subunit combinations [115, 116]. The neurotoxins lophotoxin, neosurugatoxin, erysodine, *α*-BgT, and the alkaloids DH*β*E are competitive nAChR antagonists that display selectivity for *β*2-containing nAChRs, particularly the *α*4*β*2 subtype [117]. The latest study implied that nAChR antagonists can be used for anticancer drugs. While the *α*7-nAChRs are overexpressed in small-cell lung carcinoma in smokers [118], *in vitro* experiments have suggested that the malignant growth can be ceased using snake neurotoxins (*α*-neurotoxins) or snail conotoxins (*α*-conotoxins), and these have been used for the isolation and biochemical characterization of nAChRs because they are competitive antagonists of the nAChR [119]. The presence of *α*7-nAChR inhibitors, such as methyllycaconitine (MLA) and *α*-bungarotoxin, was found to have reversed the proangiogenic effects of nicotine during cancer development process [50–52, 54]. Russo and colleagues demonstrated that several natural compounds significantly inhibited NSCLC cell proliferation or tumor growth by inhibition of *α*7-nAChR expression. These data determined a significant reduction of tumor growth in nude mice orthotopically engrafted with A549-luciferase cells (4.6% of living cells versus 31% in untreated mice). The specific *α*7-nAChR antagonists can undergo both induction of apoptosis protein (activates caspases 3, 9, 2, P53, and Bad) and reduction of survival signaling (activates PI3K-Akt, MAPK, and NF-*κ*B pathways) in *in vitro* and *in vivo* experiments. These data suggested that *α*7-nAChR-targeted chemicals form a promising prospective in anticancer drug development. However, it must be noted that both *α*-bungarotoxin and MLA also bind in high affinity to *α*9-nAChR. Therefore, there may be partial involvement of *α*9-nAChR in the proangiogenic effects of nicotine [120, 121].

Recently, the involvement of *α*9-nAChR in pain has been suggested by a number of experimental observations, and the administration of nAChR agonists reduces pain-related behaviors in several studies [122–124]. Virus-mediated overexpression of the *α*9-nAChR subtype was specifically found in breast cancer tumors [26]. Rather than using competitive nAChR inhibitors, nature compounds were investigated and shown to have inhibited cancer cell proliferation. For example, a very low concentration of garcinol (1 *μ*M) from the edible fruit *Garcinia indica *inhibited nicotine-induced breast cancer cell proliferation through the downregulation of *α*9-nAChR and cyclin D3 expression [27]. Other natural compounds, such as *luteolin* and *quercetin*, have also inhibited human breast cancer cell proliferation through the downregulation of cell surface *α*9-nAChR subunit expression in human breast cancer cells, and the combined treatment of cells with *luteolin* and *quercetin* synergistically inhibited AKT activation [28]. In another study, Tu et al. found that estradiol- and nicotine-induced *α*9-nAChR protein expression was blocked by* epigallocatechin-3-gallate *(EGCG) [125]. These findings suggested a possible chemopreventive ability of EGCG through the inhibition of estrogen- or nicotine-induced *α*9-nAChR protein expression, which is known to confer smoking-mediated breast tumorigenesis. All of these findings have provided molecular evidence for the possible chemopreventive or chemotherapeutic ability of smoking-mediated breast tumorigenesis. As always, a balance of regulating nAChR activity must be maintained between limiting pathological angiogenesis and causing potential toxicity to patients.

## 9. Conclusion

Epidemiological and experimental studies targeting nAChRs have clearly established that tobacco products cause cancers of various types. An improvement of understanding towards any relevant carcinogenic mechanisms will lead to new approaches for cancer prevention. Over the past two decades, several valuable tobacco carcinogen biomarkers have been discovered, which increases our insight into the mechanism of cancer induction. The multiple tumor-promoting effects caused by cigarette smoke and the carcinogens and toxicants in it must be targeted. The ideal drug to target these effects must have minimal toxicity in animal models and humans, which might be achievable through using naturally occurring compounds in doses no greater than those present in common foods, such as vegetables, to maintain homeostasis in the human body. At present, the majority of compounds under investigation are either agonists or partial agonists. Given the negative effects of nicotine on the immune system function, receptor subtype-selective antagonists might also be beneficial as therapeutic agents. The presence of nAChRs in tissues, in addition to the central and peripheral nervous systems, for example, immune system, gastrointestinal tract, lung, breast, and bladder, could offer additional therapeutic targets for receptor subtype-selective nAChR ligands when these become available.
